# Management of Gastric Precancerous Lesions and Early Cancer: Practice-Oriented Answers to Clinical Questions

**DOI:** 10.3390/cancers18142276

**Published:** 2026-07-15

**Authors:** Cecilia Capelli, Alberto Gattuso, Roberta Grosso, Marco Di Marco, Leonardo Frazzoni

**Affiliations:** 1 Department of Medical and Surgical Sciences, University of Bologna, 40138 Bologna, Italy; cecilia.capelli3@studio.unibo.it (C.C.); alberto.gattuso@studio.unibo.it (A.G.); roberta.grosso3@studio.unibo.it (R.G.); 2 Gastroenterology and Digestive Endoscopy Unit, Rimini Hospital, AUSL Romagna, 47923 Rimini, Italy; marco.dimarco@auslromagna.it

**Keywords:** gastric precancerous conditions, gastric precancerous lesions, early gastric cancer, endoscopic mucosal resection, endoscopic submucosal dissection, optical diagnosis, gastroscopy

## Abstract

**Background/Objectives:** Gastric precancerous conditions and early gastric cancer represent a heterogeneous disease spectrum with variable malignant potential and complex management pathways. Despite well-established international guidelines, discrepancies remain between recommended strategies and routine clinical practice, particularly regarding endoscopic diagnosis, risk stratification, therapeutic selection, and follow-up. This review aims to synthesize current evidence and provide practice-oriented, question-based guidance for the management of gastric precancerous lesions and early gastric cancer. **Methods:** A comprehensive review of the literature was conducted using PubMed and Google Scholar, focusing on endoscopic diagnosis, histological risk assessment, therapeutic options, and surveillance strategies for gastric precancerous lesions and early gastric cancer. Key areas of clinical uncertainty and controversy were identified and translated into focused, practice-oriented clinical questions designed to reflect and possibly help to improve real-world gastroenterological practice. **Results:** Clinical questions were formulated to cover the entire management pathway, from endoscopic detection and characterization to therapeutic decision-making and post-treatment surveillance. Topics include high-quality endoscopic diagnosis, biopsy strategies, histological staging system, selection between endoscopic and surgical therapy, and follow-up according to individual risk profile. For each question, current evidence is summarized into concise, actionable recommendations. **Conclusions:** Management of gastric precancerous lesions and early gastric cancer requires a structured and individualized approach, integrating high-quality endoscopy, accurate histological risk stratification, and evidence-based therapeutic and surveillance strategies. Organizing available evidence into practice-oriented clinical questions may help harmonize clinical practice, reduce unwarranted variability, and support gastroenterologists in delivering optimal patient-centered care.

## Simple Summary

Gastric cancer typically arises from precursor conditions in the stomach lining, yet significant discrepancies persist between established guidelines and routine clinical practice. To bridge this translational gap, we aimed to develop a practice-oriented framework structured around fundamental clinical questions. This review synthesizes current evidence to provide actionable recommendations for the detection, risk stratification, and treatment of early gastric abnormalities. We detail optimal techniques for high-quality endoscopic evaluation, targeted tissue sampling, and therapeutic decision-making between minimally invasive, organ-preserving resection and radical surgery. By consolidating complex literature into pragmatic guidance, this work seeks to harmonize clinical practice and reduce unwarranted therapeutic variability. Ultimately, these findings empower gastroenterologists to deliver optimal, patient-centered care, preventing disease progression while minimizing unnecessary surgical interventions.

## 1. Introduction

Gastric cancer remains one of the most lethal malignancies worldwide despite a global decline in incidence attributable to Helicobacter pylori eradication and improved surveillance. The transition from healthy mucosa to invasive carcinoma is not abrupt but follows a well-characterized multistep progression, the Correa cascade: chronic active gastritis, chronic atrophic gastritis (CAG), intestinal metaplasia (IM), dysplasia, and ultimately invasive adenocarcinoma. Each step represents both a clinically relevant disease stage and a potential window for intervention. Despite internationally endorsed guidelines, considerable heterogeneity persists in daily practice—in the quality of endoscopic examinations, the use of standardized biopsy protocols, the application of risk-staging systems such as OLGA and OLGIM, and the selection of therapy for early neoplastic lesions. This gap translates into delayed diagnoses, missed opportunities for organ-preserving treatment, and sometimes inappropriate escalation to surgery for lesions amenable to curative endoscopic resection.

Early gastric cancer (EGC)—a neoplasm confined to the mucosa or submucosa, irrespective of lymph node status—is increasingly amenable to curative endoscopic resection when detected early. Endoscopic submucosal dissection (ESD) is the gold standard for en bloc, histologically complete resection, enabling accurate staging and reducing the need for radical gastrectomy. However, achieving a curative outcome requires not only technical skill but also a structured pathway of high-quality detection, reliable optical characterization, risk stratification, and well-defined post-resection surveillance. To address these challenges, we synthesize current evidence into a practical, question-based framework. By translating key controversies into focused, practice-oriented questions, this review aims to bridge guideline recommendations and daily reality, providing actionable guidance across the full spectrum of gastric precancerous conditions and early gastric cancer.

## 2. Materials and Methods

We conducted a comprehensive review based on an independent literature search by three authors, searching PubMed and Google Scholar up to April 2026 for studies on gastric precancerous lesions and early gastric cancer—endoscopic detection and characterization, biopsy strategies, histopathological risk staging, therapeutic decision-making, and post-treatment surveillance.

The PubMed search was conducted using a combination of MeSH terms and free-text keywords: (“Stomach Neoplasms”[MeSH] OR “Gastric Neoplasms”[MeSH] OR “early gastric cancer” OR “gastric dysplasia” OR “gastric precancerous lesions” OR “gastric intestinal metaplasia” OR “chronic atrophic gastritis”) AND (“Endoscopy, Gastrointestinal”[MeSH] OR “Endoscopic Mucosal Resection”[MeSH] OR “Endoscopic Submucosal Dissection”[MeSH] OR “endoscopic resection” OR “ESD” OR “EMR” OR “chromoendoscopy” OR “narrow band imaging”) AND (“risk stratification” OR “OLGA” OR “OLGIM” OR “surveillance” OR “optical diagnosis” OR “Helicobacter pylori” OR “curative resection”). The Google Scholar search was broader, capturing guidelines and consensus documents not indexed in MEDLINE; reference lists were screened for additional articles.

Eligible studies were systematic reviews, meta-analyses, randomized controlled trials, prospective and retrospective cohort studies, and large case series in patients with gastric precancerous conditions or early gastric cancer; only peer-reviewed full-text articles were considered. We excluded single case reports, small case series (<5 patients), narrative reviews without original data, editorials, and non-human studies. No date or language restriction was applied.

The most clinically relevant and controversial aspects of management were then identified and translated into focused, practice-oriented questions reflecting real-world decision-making. Each was addressed by appraising the evidence and integrating it with current guidelines and expert interpretation to provide pragmatic, directly applicable answers for daily practice.

## 3. Results

We formulated 13 practice-oriented questions addressing the key steps in the management of gastric precancerous lesions and early gastric cancer. These questions span the comprehensive diagnostic–therapeutic pathway, including diagnostic quality and risk stratification, biopsy strategy and histological assessment, and post-treatment management. For each question, current evidence was synthesized to offer concise, evidence-based answers aimed at guiding practical endoscopic decision-making. Questions and answers are summarized in Table 1 and Table 2.

### 3.1. Diagnosis & Risk Stratification

#### 3.1.1. How Should High-Quality Endoscopy Be Performed to Optimize Detection of Gastric Precancerous Lesions and Early Gastric Cancer?

Accurate detection and characterization of precancerous lesions and EGC underpin therapeutic decision-making and depend on mucosal visibility, since thick mucus, pooled fluids (saliva/bile), and air bubbles obscure the visual field [1]. Mucolytic and defoaming agents (e.g., simethicone with N-acetylcysteine) given 20–30 min beforehand improve mucosal cleansing [2,3]. Under the 2025 ESGE guidelines, documenting mucosal cleanliness with a validated scale (e.g., GRACE, PEACE, or Barcelona) is now a mandatory performance measure [1]. Consequently, inadequate visibility (e.g., a PEACE or GRACE ≤ 1) necessitates active intraprocedural washing to avoid suboptimal inspection. Adequate gas insufflation distends the gastric wall and eliminates blind spots where lesions could be missed [4]. Beyond visibility, the ESGE recommends a 20 min slot per procedure, with a minimum observation time of ≥7 min for meticulous inspection [1]. Systematic photodocumentation also correlates with higher detection: ESGE advises at least 10 baseline photos [1], and 20-image training protocols increased EGC detection from 0.2% to 2.3% [5]. A detailed discussion regarding the specific diagnostic tools, as well as the targeted and non-targeted tissue sampling procedures for conducting high-quality endoscopy, is provided in the subsequent sections of this review. Finally, AI-based Computer-Aided Detection (CADe) systems act as real-time concurrent observers, highlighting subtle abnormalities, reducing perceptual errors, and standardizing EGC recognition across expertise levels [6]. As a rapidly emerging tool in clinical practice, AI-assisted endoscopy not only improves the early detection of gastric lesions and reduces diagnostic dependence on the operator’s experience, but also bridges the gap toward data-driven precision medicine [7].

#### 3.1.2. How to Identify Patients at Higher Gastric Cancer Risk in Endoscopy Practice?

The Operative Link on Gastritis Assessment (OLGA) and the Operative Link on Gastric Intestinal Metaplasia (OLGIM) systems assess the severity and extent of atrophy and intestinal metaplasia to identify patients at higher gastric cancer risk. Both OLGA and OLGIM require gastric biopsies per the updated Sydney protocol (two from the antrum—lesser and greater curvature, 2–3 cm from the pylorus; two from the corpus—lesser curvature ~4 cm proximal to the angle, and greater curvature ~8 cm distal to the cardia; one from the incisura angularis—angle): OLGA grades glandular atrophy [8], while OLGIM does the same for intestinal metaplasia [9]. Combining antral and corpus scores (none [0], mild [I], moderate [II], or severe [III] atrophy or IM), both systems classify patients into five stages (0 to IV) [10].

**Figure 1:**
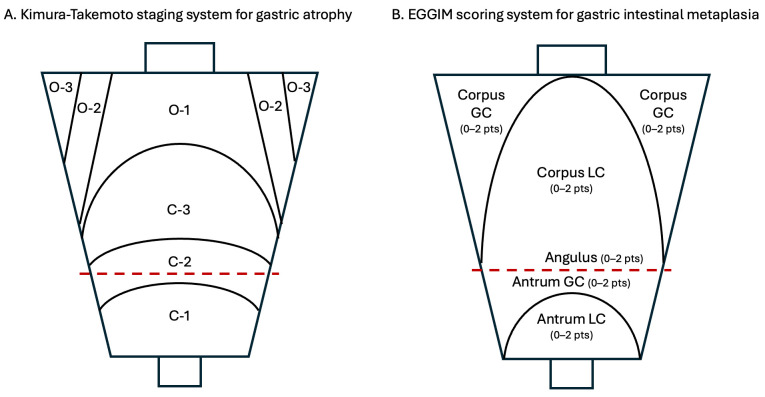
(**A**) Kimura–Takemoto staging system for gastric atrophy; (**B**) EGGIM scoring system for gastric intestinal metaplasia.

OLGA was the first staging attempt but is largely confined to research, owing to suboptimal interobserver agreement in assessing glandular loss (atrophy) [9]. It is also orientation-dependent and less reproducible [9]. OLGIM, based on intestinal metaplasia, is less orientation-dependent [11]. A newer OLGIMA system integrates both GA and IM severity per the Updated Sydney consensus. This framework includes all OLGIM III-IV patients and upstages OLGIM 0-II cases with advanced atrophy [12] (see Table 2). Advanced stages (OLGA/OLGIM III or IV) carry significantly increased gastric cancer risk. These patients warrant high-quality endoscopy every three years (1–2 years with a first-degree family history of gastric cancer) [13]. For OLGA/OLGIM 0-II, periodic surveillance is not recommended unless other risk factors are present (family history of GC, incomplete IM, persistent Helicobacter pylori infection) [14]. In practice, these systems should be integrated with the Kimura–Takemoto and EGGIM scores (see Figure 1), which endoscopically assess atrophy and metaplasia. In experienced hands, this may reduce the need for extensive biopsies—particularly with previously documented advanced stages, where random biopsies may be unnecessary at surveillance if VCE shows no visible lesion [15].

**Table 2:** Comparative analysis of gastric cancer risk stratification systems: OLGA, OLGIM, and OLGIMA [12].

Feature	OLGA	OLGIM	OLGIMA
Primary Histological Focus	Glandular Atrophy (GA) calculated as compartmental averages	Intestinal Metaplasia (IM) only	Highest individual score of either GA or IM
Staging Basis	Compartmental averages across mucosal sites	OLGIM Staging Grid (IM focus)	Maximum severity score at any topographical site
Histological Grading System	0–3 Updated Sydney Scale	0–3 Updated Sydney Scale	0–3 Updated Sydney Scale
Topographical Areas Assessed	Antrum/Incisura and Oxyntic Mucosa (Corpus)	Antrum/Incisura and Oxyntic Mucosa (Corpus)	Antrum/Incisura and Oxyntic Mucosa (Corpus)
Reproducibility among Pathologists	Low to moderate	High	High
Sensitivity for High-Risk Identification	Not established against gold standard	69.2% (Detected 2.7% prevalence of 3.9% high-risk cohort)	100% (Detected all 3.9% high-risk prevalence)
Calculated Accuracy	Not established against gold standard	98.8%	100%

#### 3.1.3. When and How Should Biopsies Be Performed in Suspected Gastric Precancerous Conditions?

Before biopsy, the mucosa should be systematically assessed with VCE, as BLI and NBI enhance visibility of atrophy and IM—the latter appearing as whitish or slightly elevated areas with a villous surface pattern [16,17]. For such conditions, biopsy sampling is recommended to risk-stratify individuals, to guide surveillance, and to detect neoplasia early, when curative resection is still achievable [13]. At least two biopsies should be taken from the antrum/incisura and two from the corpus, guided by VCE and placed in separate vials so the pathologist can determine the topographical extent of atrophy or metaplasia (OLGA/OLGIM staging). Targeted biopsies (at least two) must also be taken from any macroscopically suspected neoplastic lesion [13]. Additional random biopsies may help when no visible lesions are present or for operators less familiar with the visual features of precancerous conditions [18]. The incisura angularis biopsy is optional: although IM is most severe and frequent there, the additional biopsy yields only a small absolute increase in patients staged OLGA/OLGIM III/IV [19,20]. Therefore, the additional incisura angularis biopsy should be considered if it is not possible to perform an adequate endoscopic evaluation of the mucosa using VCE [21]. Additional tissue specimen(s) should be taken to assess the Helicobacter pylori (HP) status, as its prompt eradication is crucial to prevent precancerous progression and reduce malignancy risk [22]. Any suspicious abnormality should be biopsied separately into its own labeled jar after adequate description (size, Paris morphology, location, patterns) and photodocumentation. Early lesions warrant two biopsies, which do not compromise subsequent resection, whereas advanced lesions require six [23]. If random biopsies detect dysplasia (or indefinite for dysplasia), a high-quality endoscopy should be repeated, using VCE if not done before, given its superior diagnostic yield [24]. If still no lesion is seen, a further EGD with WLE and VCE (or dye-based chromoendoscopy) with guided biopsies is needed: at 6 months for HGD and 12 months for LGD/indefinite for dysplasia [13].

#### 3.1.4. Which Endoscopic Features Raise Suspicion for High-Risk Gastric Precancerous Lesions or Early Cancer?

Identifying high-risk precancerous lesions—low-grade (LGD) and high-grade dysplasia (HGD)—requires a thorough assessment of the background mucosa. Dysplasia rarely arises in healthy mucosa, typically developing in a high-risk microenvironment altered by CAG and GIM [13]. Under white-light endoscopy (WLE), key features of dysplasia and EGC include dyschromia, focal loss of vascularity, subtle elevations or depressions, nodularity, mucosal thickening, and abnormal convergence or flattening of gastric folds [25].

**Figure 2:**
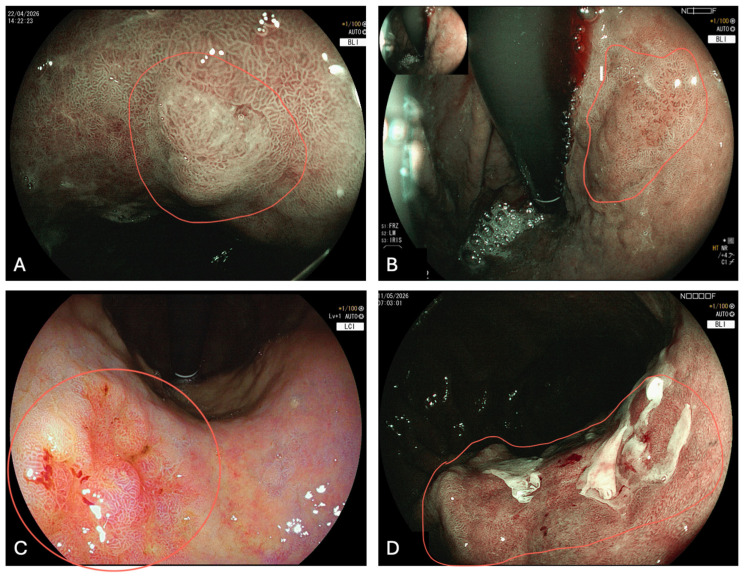
(**A**) Gastric low-grade dysplasia; (**B**) Gastric high-grade dysplasia; (**C**) Early gastric cancer; (**D**) Invasive gastric cancer.

Distinguishing grades is crucial: HGD carries a substantial risk of progression to intramucosal carcinoma and often shows high-risk macroscopic features [25], such as larger size (typically >2 cm), depressed morphology, marked erythema reflecting tumor neoangiogenesis, and highly irregular yet sharply demarcated margins [26,27]. Conversely, LGD usually appears as a flat or slightly elevated lesion with whitish discoloration blending into the surrounding mucosa [26,27]. The macroscopic appearance of dysplasia and EGC is standardized by the Paris Classification, which stratifies superficial (Type 0) lesions into polypoid (0-I), flat/elevated (0-IIa, 0-IIb), depressed (0-IIc), or ulcerated (0-III) subtypes [28]. The Type 0-IIc variant is particularly important, accounting for 70–80% of EGCs and strongly predicting underlying HGD or mucosal carcinoma [29]. Specific WLE markers further raise suspicion for EGC. Spontaneous bleeding or marked friability without trauma, a highly specific sign of neoangiogenesis, is seen in over half of EGC lesions [30,31]. Severe atypia produces a rough surface that distorts or fragments the normal specular light reflection under WLE—a feature suspicious for type 0-IIb or 0-IIc EGC [30,32]. Finally, EGCs show sharp chromatic boundaries: differentiated intestinal-type tumors appear as well-demarcated erythematous areas with spontaneous bleeding, whereas undifferentiated diffuse-type tumors present as flat or depressed zones of marked pallor (see Figure 2) [4].

#### 3.1.5. What Is the Role of Virtual Chromoendoscopy and Magnification in Gastric Precancerous Conditions and Lesions Characterization?

**Figure 3:**
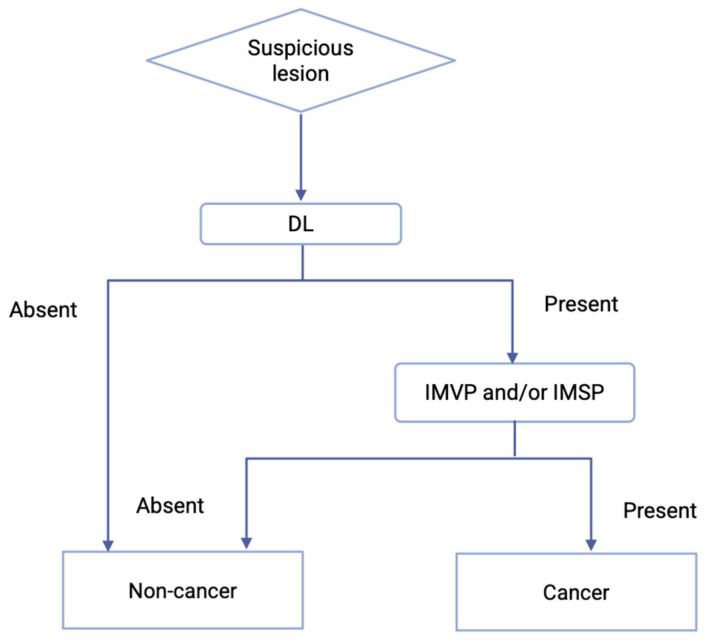
MESDA-G algorithm. DL, demarcation line; IMVP, irregular microvascular pattern; IMSP, irregular microsuperficial pattern.

WLE has historically been the standard, but its suboptimal sensitivity (33–75%) for early gastric cancer (EGC) has driven a transition toward Image-Enhanced Endoscopy (IEE) modalities, specifically virtual chromoendoscopy (VCE) and magnifying endoscopy (ME) [33]. The MAPS III guidelines now mandate routine high-quality endoscopy incorporating VCE [13]. VCE modalities—Narrow-Band Imaging (NBI), Blue Laser Imaging (BLI), and Linked Color Imaging (LCI)—use narrowed spectral transmittance and color enhancement to heighten contrast between the mucosal surface and subepithelial microvascular networks. Meta-analyses show that routine VCE improves detection and diagnostic precision for both EGCs (85% vs. 56.7%) and preneoplastic conditions (88.9% vs. 40.1%) versus WLE [34]. While VCE provides macroscopic contrast enhancement, ME uses a motorized lens for 80–100× magnification. Combining ME with VCE (e.g., ME-NBI and ME-BLI) enables an “optical biopsy” of mucosal crypts and capillary networks [35]. This identifies optical markers of gastric intestinal metaplasia (GIM) such as Light Blue Crests (LBCs) and the White Opaque Substance (WOS) [36]. LBCs are thin, continuous or crested lines of brilliant light blue color, usually arranged along the superficial margins of the foveolar glandular structure, whereas WOS is accumulated lipid droplets obscuring subepithelial capillaries [37,38]. The Vascular Surface (VS) Classification characterizes superficial lesions and differentiates neoplastic from non-neoplastic tissue using ME and VCE. It defines EGC by a clear demarcation line (DL) between cancerous and non-cancerous mucosa with an irregular microvascular (MV) and/or microsurface (MS) pattern—features present in 97% of EGCs [39]. It yields high diagnostic accuracy (79% to >95%), with sensitivity and specificity up to 95% and 96%, respectively [40,41]. However, flat, discolored, undifferentiated-type lesions remain a recognized limitation [29,40]. Building on this, the Magnifying Endoscopy Simple Diagnostic Algorithm for Early Gastric Cancer (MESDA-G) provides a consensus for EGC diagnosis (Figure 3) [42,43,44]. MESDA-G first assesses the DL: its absence classifies the lesion as non-neoplastic; if present, the internal MV and MS patterns are evaluated [43,45]. It achieves ~95% diagnostic accuracy and a 99% negative predictive value, though its efficacy for diffuse-type EGC remains insufficiently characterized, highlighting the need for future targeted investigations [43].

#### 3.1.6. When Is Endoscopic Surveillance Sufficient for Gastric Precancerous Conditions?

Surveillance monitors individuals at elevated risk of gastric adenocarcinoma without an immediate resection indication. For atrophy and intestinal metaplasia, it is indicated at advanced stages, defined by extensive antral and corpus involvement or OLGA/OLGIM III to IV [13,46]. Endoscopically, these advanced stages correlate with Kimura-Takemoto scores of C3 or higher, or EGGIM scores of 5 or higher [13,47]. Surveillance is recommended every three years, intensified to 1–2 years with aggravating factors, notably a first-degree family history of gastric cancer [13,48]. For LGD, HD-WLE with VCE is recommended strictly when no endoscopically visible lesion is present [13]. If reassessment confirms no discernible focal lesion, monitoring at 12-month (6-months in case of HGD) intervals is advised to detect progression [13]. Surveillance is no longer appropriate once dysplasia of any grade or EGC presents as a visible lesion [13]. In such scenarios, the standard of care shifts to formal staging followed by endoscopic resection [13]. ESD—or EMR for select small non-ulcerated lesions—then provides definitive histopathological staging and potential curative resection, replacing observation with active intervention [13,48].

#### 3.1.7. Which Lesions Are Best Managed with Endoscopic Resection Rather than Surveillance?

The choice between surveillance and resection for gastric dysplasia depends on macroscopic visibility and histological grade. Resection is indicated exclusively for visible dysplastic lesions, whether low-grade (LGD) or high-grade (HGD) [13]. It both achieves curative resection and provides the entire specimen for accurate staging, overcoming the sampling errors of forceps biopsies. Conversely, when dysplasia or “indefinite for dysplasia” is found on random biopsies without a visible lesion, blind prophylactic resection is contraindicated; guidelines instead mandate a stepwise protocol [13]:

- Pathological revision: The initial biopsy should be reviewed by an expert gastrointestinal pathologist to confirm the diagnosis.
- Second endoscopic evaluation: A high-quality “second-look” with HD-WLE and VCE to search for the missed lesion, map background precancerous conditions, and perform HP testing.
- Strict surveillance: If the lesion remains occult, repeat endoscopy within 6 months for invisible HGD (high risk of concurrent EGC) or 12 months for invisible LGD or indefinite findings.

### 3.2. Therapeutic Decision-Making

#### How Should the Choice Between EMR and ESD Be Made for Early Gastric Neoplasia?

Curative endoscopic resection for EGC relies on precise preoperative staging and LNM-risk stratification, using tools such as the eCura system [49]. High-definition endoscopy assessing size, Paris morphology, and mucosal/vascular patterns is required to estimate invasion depth and resectability [13]. Both Japanese and European guidelines now recommend ESD as the primary treatment for most superficial gastric lesions [13,50]. EMR is primarily restricted to elevated, non-ulcerated lesions ≤ 10 mm with a low risk of malignancy [13]. Reliable en bloc resection with EMR is difficult for lesions > 15 mm, and the resulting piecemeal specimens compromise staging and increase local recurrence [51,52]. ESD achieves significantly higher en bloc (OR = 4.00, *p* < 0.00001) and R0 curative resection (OR = 1.95, *p* < 0.00001) than EMR [51]. Absolute indications include differentiated, non-ulcerated intramucosal carcinomas of any size, and ulcerated lesions ≤ 30 mm [13,50]. Expanded criteria allow ESD for differentiated lesions with superficial submucosal invasion (≤500 μm) measuring ≤30 mm, and non-ulcerated undifferentiated intramucosal lesions ≤ 20 mm [13]. ESD also lowers local recurrence (OR = 1.97, *p* < 0.00001) [51,52], especially in differentiated lesions (OR = 3.85, *p* < 0.001) [51,52]. Another crucial factor influencing the choice between ESD and EMR is the presence of submucosal fibrosis, which can be due to prior ulceration, chronic inflammation, or previous biopsies [53,54]. Because fibrotic tissue restricts fluid expansion during injection, it creates a ‘non-lifting’ sign that makes standard EMR unsafe and prone to incomplete excision [55]. To address this limitation, European guidelines support ESD for most superficial gastric lesions [55], while Japanese guidelines strictly require ESD to ensure en bloc resection of ulcerated lesions [54]. However, the safety profile dictates caution: ESD involves prolonged procedural times and a significantly higher perforation risk than EMR (OR = 7.90, *p* < 0.0001) [51]. Decisions should be multidisciplinary, balancing oncological efficacy against comorbidities, center volume, and operator expertise [13,51].

### 3.3. Post-Treatment Management

#### 3.3.1. Which Endoscopic and Histological Features Define Curative Endoscopic Resection of a Gastric Superficial Neoplasm?

According to ESGE guidelines, curative endoscopic resection requires achieving en bloc excision with microscopically negative lateral (horizontal) and deep (vertical) margins, absence of lymphovascular invasion (LVI), and a histopathologically confirmed LNM risk < 1%, rendering the endoscopic approach oncologically equivalent to surgical gastrectomy [13]. A “very low risk” LNM profile is met by dysplasia only, or well-differentiated pT1a lesions of any size if non-ulcerated or ≤30 mm if ulcerated. For undifferentiated histology, curability requires pT1a, size < 20 mm, and absence of ulceration. Since neoplasms confined above the muscularis mucosae carry an LNM risk approaching 0%, they are the ideal candidates for curative endoscopic eradication [56]. ESGE, ASGE, and JGCA categorize these curative resections differently. The ESGE employs a four-tier system (very-low, low, local, and high risk) that restructures the traditional Japanese absolute/expanded distinction [13]. ESGE very-low risk merges the JGCA absolute criteria (eCura A) with most expanded criteria for differentiated pT1a—removing the 20 mm size limit for non-ulcerated differentiated mucosal cancers and including ulcerated differentiated pT1a ≤ 30 mm (JGCA eCura B) [50]. ESGE low risk is reserved for the two subtypes with higher LNM risk (~3%): differentiated pT1b SM1 (≤500 µm, ≤30 mm) and undifferentiated pT1a (≤20 mm, no ulceration) [13]. The ASGE does not adopt formal risk-tier nomenclature but endorses the Japanese absolute and expanded criteria as a unified framework, stratifying surveillance by depth of invasion (T1a vs. T1b) rather than a named risk category [57]. The JGCA eCura system maintains the absolute (eCura A) vs. expanded (eCura B) distinction, with eCura B covering all expanded criteria despite heterogeneous LNM risk (0.27% for differentiated pT1a > 20 mm to 2.6% for pT1b SM1) [50].

#### 3.3.2. When Should Surgery Be Recommended After Endoscopic Resection of Early Gastric Cancer?

**Figure 4:**
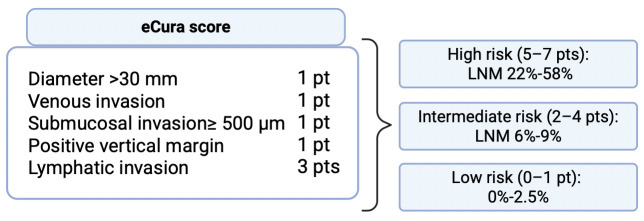
eCura scoring system for quantifying lymph node metastasis potential. LNM, lymph node metastasis.

Although endoscopic resection is the gold standard for EGC, about 18% of resections in Asian high-volume centers and 23–25% in Western series fail to meet curative criteria, mandating subsequent surgery [54,55,58]. This decision balances the individual risk of occult regional LNM against the morbidity of major gastric surgery. According to ESGE guidelines, non-curative resections are stratified based on the biological risk of lymph node dissemination. A resection is classified as “local risk” when curative criteria are unmet solely due to a positive horizontal margin or a piecemeal resection. Since the LNM risk in these specific scenarios remains exceptionally low (<3%), guidelines favor conservative management over radical surgery. The recommendation is to perform close endoscopic surveillance combined with repeat ESD or argon plasma coagulation (APC) to manage any residuals or recurrences. Conversely, “high risk” resections carry a substantial risk of systemic dissemination: a positive vertical margin, lymphovascular invasion (LVI), or deep submucosal invasion (>500 μm). It also applies to poorly differentiated lesions that are ulcerated or >20 mm, differentiated lesions with superficial submucosal invasion (≤500 μm) > 30 mm, or intramucosal ulcerative lesions > 30 mm. Any of these markers raises occult LNM risk beyond the 3% threshold [13]. A salvage gastrectomy with regional lymph node dissection should then be considered [13]. Meta-analyses show that salvage gastrectomy after a high-risk non-curative resection markedly improves long-term outcomes, including 5-year overall survival (OR 3.63, HR 0.40) and disease-free survival (OR 4.39) [59]. To precisely quantify LNM potential and refine surgical indications, clinicians can employ the eCura scoring system [60,61], illustrated in Figure 4. It assigns points to adverse factors, weighting lymphatic invasion (3 points) and tumor size, submucosal depth, venous invasion, and vertical margin (1 point each). Scores of 0–1 indicate low LNM risk (~2.5%) and allow observation, whereas 5–7 indicate severe risk (~22.7%) requiring radical surgery [60]. For undifferentiated histology, guidelines diverge: the ASGE prioritizes surgery [57]; both European (MAPS III) and Japanese guidelines offer conditional expanded indications for undifferentiated intramucosal lesions ≤ 20 mm without ulceration, permitting strict endoscopic follow-up in highly selected patients provided en bloc resection with negative margins was achieved [13,50,54]. However, any salvage gastrectomy decision must be evaluated within a multidisciplinary team (MDT) discussion accounting for age, comorbidities, and life expectancy. For elderly patients with significant comorbidities, vigilant non-operative endoscopic follow-up should be considered, even with a high-risk pathology report [13,62].

#### 3.3.3. What Is the Optimal Endoscopic Follow-Up After Resection of Gastric Superficial Neoplasms?

ESD achieves high curative resection rates for superficial gastric neoplasms, from low-grade adenomas to early gastric cancer [63]. However, the preserved gastric mucosa leaves a persistent risk of metachronous lesions [64]. Cumulative metachronous lesion incidence is 9.4% after endoscopic resection, peaking three to seven years afterward [65]. Local recurrence is very low (0.4%) after complete en bloc resection but rises significantly after piecemeal procedures [66]. Continuous surveillance is therefore mandated by European (ESGE), American (ASGE), and Japanese (JGES) guidelines to detect local recurrence and metachronous neoplasia, with follow-up frequency based on histological risk [13,50,54,55,57].

- For very-low risk/eCura A resections, all guidelines agree that no additional treatment is needed and endoscopic surveillance is sufficient.
- For low-risk resections (pT1b sm1 and undifferentiated pT1a), the ESGE mandates complete staging with CT and MDT discussion before choosing surveillance versus additional treatment—a requirement absent from the ASGE and JGCA. The ASGE recommends cross-sectional imaging (CT and/or EUS) every 6–12 months for 3–5 years for T1b sm1 lesions without mandating MDT review, while the JGCA considers all eCura B resections curative and recommends surveillance without additional treatment or MDT discussion.
- The ESGE local-risk category (piecemeal or HM1 resection with VM0, no LVI, all other criteria met) recommends endoscopic retreatment (re-ESD or ablation) over surgery at 3–6 months post-ESD, with scar biopsies at every follow-up. The ASGE likewise accepts additional endoscopic therapy within 3–6 months in lieu of surgery when a positive lateral margin is the sole non-curative criterion, with biopsy sampling for piecemeal resection or positive margins. The conceptually equivalent JGCA eCura C-1 category is less prescriptive, recommending either “additional treatment or close surveillance.”

#### 3.3.4. Is There a Role for *H. pylori* Eradication After Endoscopic Resection of Superficial Neoplasms?

HP is a Group 1 carcinogen driving the Correa cascade from chronic gastritis to atrophy, IM, dysplasia, and adenocarcinoma [67]. Although ESD effectively treats EGC and precancerous lesions, the preserved background mucosa remains susceptible to metachronous gastric cancer (MGC) [68,69]. A landmark trial by Choi et al. and recent meta-analyses demonstrate that post-ESD HP eradication reduces long-term MGC incidence by over 50% [70]. Eradication halves the risk of a second primary tumor even in stomachs with established structural damage [71,72]. It resolves chronic inflammation and promotes regression of atrophy, particularly in patients under 70 [71]. Meta-analyses confirm it lowers metachronous lesion incidence to levels comparable to naturally uninfected individuals [73,74]. Consequently, prompt HP eradication offers important long-term protection and should be recommended for all patients treated for EGC or precancerous conditions [13,50].

#### 3.3.5. How Should Surveillance Be Tailored After Curative Endoscopic Resection of Early Gastric Cancer?

Although annual surveillance is the baseline after endoscopic resection, long-term intervals should be tailored to the individual risk profile. Unlike traditional Japanese strategies favoring uniform annual surveillance regardless of baseline atrophy stage [66], European MAPS III guidelines advocate a risk-based approach [13]. Patients with extensive endoscopic changes (e.g., EGGIM ≥ 5) or advanced histopathological stages of GAC and IM (OLGA/OLGIM III/IV) face elevated metachronous risk and need high-quality endoscopy every three years, provided there are no additional clinical risk factors [13]. Conversely, it can be safely omitted for mild, antrum-restricted atrophy without additional risk factors [13]. The FAMISH score further refines this by stratifying patients into low (0–1), intermediate (2), and high-risk (3–9 points) tiers [64]. The score evaluates six clinical predictors: family history, age >65, male sex, corpus intestinal metaplasia, synchronous lesions, and persistent HP infection [64]. Because low- and intermediate-risk patients rarely develop early metachronous lesions, their intervals may be safely prolonged [64]. This tailored approach optimizes resources and minimizes patient burden without compromising oncological safety.

**Figure 5:**
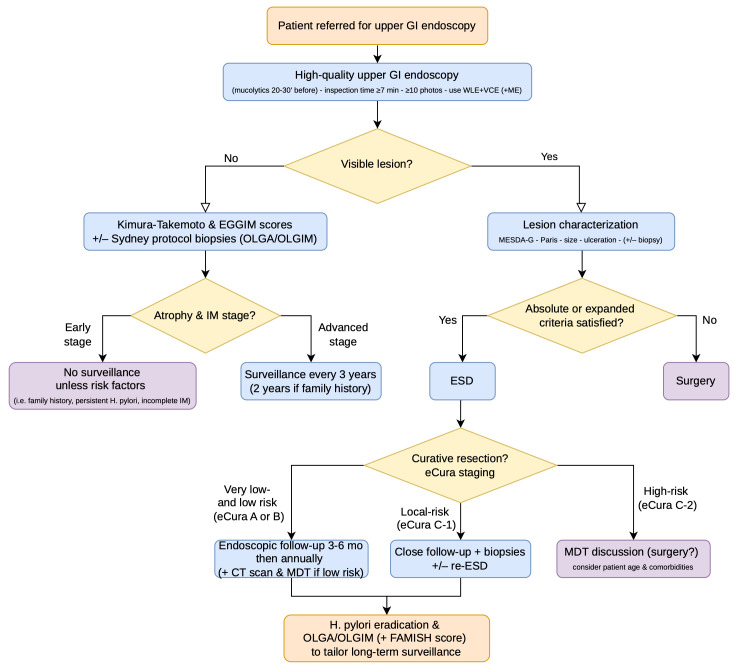
Proposed diagnostic and therapeutic algorithm for the management of gastric precancerous lesions and early gastric cancer. The flowchart integrates high-quality upper gastrointestinal endoscopy standards, risk stratification of the background mucosa (atrophy and intestinal metaplasia staging), optical characterization of visible lesions, therapeutic decision-making (ESD vs. surgery), curability assessment (eCura staging), and tailored post-resection surveillance strategies. eCura, endoscopic curability scoring system; EGGIM, endoscopic grading of gastric intestinal metaplasia; ESD, endoscopic submucosal dissection; FAMISH, family history, age, male sex, corpus intestinal metaplasia, synchronous lesions, and persistent Helicobacter pylori; GI, gastrointestinal; *H. pylori*, *Helicobacter pylori*; IM, intestinal metaplasia; MDT, multidisciplinary team; ME, magnifying endoscopy; MESDA-G, magnifying endoscopy simple diagnostic algorithm for gastric cancer; mo, months; OLGA, operative link for gastritis assessment; OLGIM, operative link on gastric intestinal metaplasia assessment; VCE, virtual chromoendoscopy; WLE, white-light endoscopy.

To synthesize the key decision points discussed in this review, we propose a comprehensive management algorithm, illustrated in Figure 5.

## 4. Discussion

Gastric cancer continues to arise predominantly through the Correa cascade [67], yet most precancerous conditions and early lesions remain potentially curable when detected and managed within a structured pathway. The present question-based synthesis highlights that optimal outcomes no longer depend on any single technique, but on the ability to integrate high-quality detection, reliable optical characterization, accurate histological risk stratification, and a tailored, organ-preserving therapeutic strategy [13,55]. The most substantial change concerns the diagnostic paradigm itself. Upper endoscopy is shifting away from the historical model of untargeted random biopsies toward an optically driven examination [13]. High-definition endoscopy combined with virtual chromoendoscopy and magnification now allows the endoscopist to recognize atrophy and intestinal metaplasia in vivo—through markers such as light-blue crests and the white opaque substance [36,37,43]—and to identify neoplasia using structured criteria such as the VS classification and MESDA-G [43,44]. When coupled with endoscopic staging surrogates (Kimura–Takemoto, EGGIM) [15] and their histological counterparts (OLGA/OLGIM) [8,10], this approach enables biopsies to be guided rather than scattered, reducing reliance on extensive random sampling while improving the detection of gastric precancerous lesions [34,35]. This paradigm change has been driven by parallel advances on two fronts. Technological refinement of endoscopes has improved mucosal visibility and lesion characterization, while the progressive dissemination of ESD to Western centers—together with the cultural attention to early detection inherited from Japanese practice—has expanded the proportion of precancerous lesions and early gastric cancers amenable to endoscopic resection [56,58]. Reflecting this convergence, both Eastern and European guidelines now position ESD as the primary treatment for most superficial gastric neoplasms [50,55], owing to its superior en bloc and R0 resection rates over EMR [51,52]. Therapeutic decision-making consequently hinges on curability. Resections meeting very low-risk criteria are oncologically equivalent to surgery, whereas low-risk resections may be considered curative after adequate post-resection staging with a CT scan and MDT discussion [55]. On the other hand, non-curative resections require risk-adapted management quantified by tools such as the eCura system [60]: local-risk cases favor endoscopic retreatment over surgery, while high-risk features mandate multidisciplinary discussion of salvage gastrectomy [59], weighed against the patient’s age, comorbidities, and life expectancy. The residual divergence between ESGE, ASGE, and JGCA frameworks—particularly for undifferentiated histology and submucosal invasion—underscores that these decisions remain best individualized within an expert, multidisciplinary setting [13,50,57]. Finally, management does not end at resection. HP eradication roughly halves the risk of metachronous neoplasms [70,73] and should be offered to all treated patients; surveillance should be tailored to the individual risk profile—using tools such as the FAMISH score [64] and OLGA/OLGIM stage—to balance oncological safety against unnecessary procedural burden [65,66].

However, transitioning to this advanced diagnostic and therapeutic paradigm presents notable implementation challenges. High-quality, optically guided endoscopy necessitates access to modern image-enhanced endoscopes and requires endoscopists to overcome a steep learning curve to reliably apply optical classifications like MESDA-G. Furthermore, the widespread adoption of ESD, particularly in Western practice, is hindered by its technical complexity, longer procedural times, and higher complication risks compared to EMR. Overcoming these clinical barriers will require structured, competency-based training programs for optical diagnosis, alongside the progressive centralization of ESD procedures in specialized, high-volume centers to ensure oncological safety and optimal patient outcomes.

This review has limitations. As a narrative, question-based synthesis, it provides a qualitative rather than quantitative appraisal of the evidence. Much of the supporting data originates from high-volume Eastern centers and may not be fully generalizable to all Western settings, and several recommendations necessarily rely on expert interpretation where high-level evidence is still maturing.

## 5. Conclusions

The management of gastric precancerous lesions and early gastric cancer increasingly relies on a structured, individualized, and evidence-based pathway that integrates high-quality optically guided endoscopy, accurate histological risk stratification, and curative organ-preserving therapy [13,55]. By translating current evidence into practice-oriented questions, this review aims to bridge the gap between guideline recommendations and everyday clinical practice, harmonize management, reduce unwarranted variability, and support endoscopists across the full disease spectrum.

## 6. Future Directions

While the current management of gastric precancerous lesions relies heavily on high-quality image-enhanced endoscopy and histological staging, emerging technologies are poised to shift the paradigm toward precision medicine.

First, the role of Artificial Intelligence (AI) is rapidly expanding beyond simple Computer-Aided Detection (CADe). According to recent guidelines, next-generation Computer-Aided Diagnosis (CADx) systems must evolve to support risk stratification by integrating endoscopic images with clinical, genomic, and epigenomic data (e.g., DNA methylation and microsatellite instability). These multi-dimensional AI models will be crucial to individualize the optimal timing for endoscopic surveillance [75].

Second, the discovery and application of novel biomarkers will increasingly rely on advanced analytical techniques coupled with Machine Learning (ML). Recent reviews highlight that direct spectroscopic methods, such as Surface-Enhanced Raman Spectroscopy (SERS) and Fourier Transform Infrared Spectroscopy (FTIR) integrated with ML, offer outstanding diagnostic accuracy, sensitivity, and specificity for cancer biomarker detection. These multi-modal approaches have the potential to outperform traditional modalities in clinical settings, allowing for highly precise, early detection [76].

Finally, the exploration of the microbiome is revealing critical non-invasive biomarkers along the oral-gut axis. Recent population-based studies have demonstrated that oral microbiome dysbiosis—particularly in subgingival sites enriched with Fusobacterium and Prevotella—is strongly associated with upper gastrointestinal precancerous conditions, including gastric atrophy and intestinal metaplasia. Evaluating these site-specific microbial signatures may soon provide a novel tool to predict malignant progression along the Correa cascade [77].

## Figures and Tables

**Table 1:** Management of gastric precancerous lesions and early gastric cancer: practice-oriented answer to clinical questions.

Clinical Question	Key Recommendation
	**Diagnosis & risk stratification**
**How should high-quality endoscopy be performed?**	Adhere to ESGE performance measures for upper GI endoscopy (clean mucosa, observation time of ≥7 min, ≥10 photos).
How to identify patients at higher gastric cancer risk?	OLGA Stages III–IV: endoscopic surveillance every 3 years (1–2 if family history). OLGA Stages 0–II: no routine surveillance is required unless other risk factors exist.
When and how should biopsies be performed in suspected gastric precancerous conditions?	Endoscopic staging (Kimura-Takemoto/EGGIM) ± random sampling to risk-stratify individuals → ≥2 biopsies from antrum/incisura and 2 from corpus in separate vials Any endoscopically visible or suspected neoplastic lesion → ≥2 targeted biopsies.
Which endoscopic features are suspected for gastric precancerous lesions or EGC?	**General hallmarks**: dyschromia, focal loss of vascularity, mucosal nodularity/thickening, abnormal convergence of gastric folds. **HGD predictors**: lesion size > 2 cm, depressed morphology, marked surface erythema, and irregular but sharply demarcated margins. **EGC markers**: spontaneous bleeding, severe mucosal friability, rough surface texture (altered specular light reflection).
What is the role of VCE and ME in gastric precancerous conditions and lesions characterization?	VCE is mandatory to enhance macroscopic contrast. ME-VCE allows “optical biopsy” → application of the MESDA-G algorithm for EGC diagnosis
	**Treatment & surveillance**
When is endoscopic surveillance appropriate for precancerous conditions?	Endoscopic surveillance is appropriate for advanced stages (OLGA/OLGIM III–IV) and LGD without a visible lesion. Once a visible lesion is identified, endoscopic resection is indicated.
Which lesions are best managed with endoscopic resection?	Any endoscopically visible dysplastic lesion (LGD or HGD) must be resected to ensure cure and accurate staging. Avoid routine blind resection for non-visible dysplasia.
Which endoscopic technique should be reserved for early gastric neoplasms?	ESD is the standard of care for optimal en bloc resection. EMR is not the standard and should be strictly reserved for 0-IIa non-ulcerated lesions ≤ 10 mm with low malignancy risk.
What defines a curative endoscopic resection of a gastric superficial neoplasm?	**Endoscopy-related factors**: en bloc resection, R0 margins (horizontal/vertical) **Lesion-related factors**: no lymphovascular invasion (LVI); ○ if well-differentiated → depth of invasion ≤ pT1a (intramucosal) or pT1b SM1 if ≤30 mm, size ≤ 30 mm if ulcerated; ○ if undifferentiated → pT1a only, size < 20 mm, no ulceration
When should surgery be recommended after endoscopic resection?	Surgery is recommended in “high-risk” non-curative resections (e.g., positive vertical margin, deep submucosal invasion, LVI+). Decisions should be guided by the eCura score and discussed within an MDT.
What is the optimal endoscopic follow-up after resection?	Annual surveillance for low-risk resections. Local-risk resections (e.g., piecemeal/margin+) require stricter initial follow-up (e.g., at 3, 6, and 12 months).
Should *H. pylori* be eradicated after endoscopic resection of gastric superficial neoplasms?	Yes, eradication is strongly recommended to reduce the incidence of metachronous neoplasms.
How should surveillance be tailored after curative EGC resection?	Personalize based on risk (e.g., FAMISH score). High-risk (OLGA/OLGIM III/IV) → 3-year surveillance. Low risk → may safely omit or extend follow-up intervals.

eCura, endoscopic curability; EGC, early gastric cancer; EMR, endoscopic mucosal resection; ESD, endoscopic submucosal dissection; ESGE, European Society of Gastrointestinal Endoscopy; FAMISH, family history, atrophy, metaplasia, intestinal phenotype, size, Helicobacter pylori; GI, gastrointestinal; HGD, high-grade dysplasia; LGD, low-grade dysplasia; LVI, lymphovascular invasion; MDT, multidisciplinary team; ME, magnifying endoscopy; MESDA-G, magnifying endoscopy simple diagnostic algorithm for gastric cancer; OLGA, operative link for gastritis assessment; OLGIM, operative link on gastric intestinal metaplasia assessment; SM, submucosal; VCE, virtual chromoendoscopy.

## Data Availability

No new data were created or analyzed in this study. Data sharing is not applicable to this article.

## Abbreviations

The following abbreviations are used in this manuscript:

AI	Artificial Intelligence
APC	Argon Plasma Coagulation
ASGE	American Society for Gastrointestinal Endoscopy
BLI	Blue Laser Imaging
CAD	Computer-Aided Detection
CAG	Chronic Atrophic Gastritis
DL	Demarcation Line
eCura	Endoscopic Curability (scoring system)
ECG	Early Gastric Cancer
EGDS	Esophagogastroduodenoscopy
EGGIM	Endoscopic Grading of Gastric Intestinal Metaplasia
EMR	Endoscopic Mucosal Resection
ESD	Endoscopic Submucosal Dissection
ESGE	European Society of Gastrointestinal Endoscopy
FAMISH	Family history, Age > 65, Male sex, corpus Intestinal metaplasia, Synchronous lesions, and persistent *H. pylori* (scoring system)
HD-WLE	High-Definition White-Light Endoscopy
HGD	High-Grade Dysplasia
HP	*Helicobacter pylori*
IEE	Image-Enhanced Endoscopy
IM	Intestinal Metaplasia
JGCA	Japanese Gastric Cancer Association
LBC	Light Blue Crest
LGD	Low-Grade Dysplasia
LNM	Lymph Node Metastasis
MDT	Multidisciplinary Team
ME	Magnifying Endoscopy
MESDA-G	Magnifying Endoscopy Simple Diagnostic Algorithm for Early Gastric Cancer
MGC	Metachronous Gastric Cancer
MGLs	Metachronous Gastric Lesions
MS	Microsurface
MV	Microvascular
NBI	Narrow-Band Imaging
OLGA	Operative Link on Gastritis Assessment
OLGIM	Operative Link on Gastric Intestinal Metaplasia
VCE	Virtual Chromoendoscopy
VS	Vascular Surface
WLE	White-Light Endoscopy
WOS	White Opaque Substance
